# Supplementary material for: DisCVR: Rapid viral diagnosis from high-throughput sequencing data
Source: Virus Evol. 2019 Aug 26;5(2):vez033. doi: 10.1093/ve/vez033 (PMC6735924; doi:10.1093/ve/vez033)
Supplement: vez033_Supplementary_Data [file vez033_supplementary_data.zip › SupplementarySectionS1_final.docx]

**Supplementary Methods for DisCVR: Rapid viral diagnosis from high-throughput sequencing data**

**Low-complexity *k*-mers**

In order to exclude low-complexity *k*-mers, DisCVR calculates the tri-nucleotide Shannon entropy values of the *k*-mers (Shannon 1948). Examples consisting of a repeated nucleotide, a repeated tri-nucleotide and a sequence having no repeated pattern had entropy values of 0, 1 and ~3, respectively, thus showing that this value is low for low-complexity *k*-mers (Supplementary Table S1). A program was written to extract *k*-mers of various sizes (*k*=18, 22, 26 or 30) from the pathogenic dataset, and their tri-nucleotide entropy values were calculated (Fig. S1). Low complexity *k-*mers were defined based on a threshold choice of ≤2.5, which excluded low-complexity *k*-mers while still including the majority of *k*-mers, with a larger proportion being retained as the size of *k* increased. This value was chosen as the default threshold setting built into DisCVR. The influence of filtering low-entropy *k-*mers is discussed below.

**Optimal size of *k***

The theoretical minimum size of a *k*-mer is the *k* size at which the largest number of *k*-mers is obtained and can be approximated by log_x_(N) where x is the alphabet size and N is the length of the largest genome. It has been shown that reliable tree topologies are typically obtained when the *k* size is greater that this threshold (Sims et al. 2009). Similarly, the optimal feature length, i.e., the *k*-mer size for which the information content of a genome can be approximated by the ensemble of its *k*-mers, can be calculated by 2log_x_(N) as shown empirically and theoretically (Sims et al. 2009; Wu et al. 2009). Thus, the theoretical range of *k*-mer size for the hemorrhagic dataset are between 5 and 10 and for respiratory dataset and the pathogenic dataset, the theoretical *k* size for classification range from 9 to 18.

The proportions of virus *k*-mers shared with the host *k*-mers were calculated for the pathogenic dataset for increasing values of *k*, in relation to the total number of possible *k*-mers (4*^k^*). The results show that the proportion of virus *k*-mers became greater than that of shared *k*-mers at *k*=18 although at *k*=18 there are still a small proportion of *k*-mers that are common between the host and virus sequences (Fig. S2). This pattern was the same after eliminating low-complexity *k*-mers. As the proportion of *k*-mers shared between the host and the viruses plateau at k=22 and the average number of hits per sample dips at *k*=22, a sign of the reduction in noise, an optimal *k*-mer size of 22 was chosen.

The pathogenic and respiratory datasets were each used to construct virus *k*-mer databases at *k*=18, 22, 26 and 30 (Fig. S3). Each database was then used to analyse the published HTS data (Thorburn et al. 2015). The results confirmed that the smaller the *k*-mer, the less time taken, and the larger the *k*-mer, the lower the number of virus hits (Fig. S3). In addition, more time was required and more hits were obtained by using the pathogenic dataset, which is larger than the respiratory dataset.

Table S3 and S4 compares the results for the 48 samples that had tested positive by RT-PCR with the top scoring classification results obtained using the four databases generated from the respiratory dataset and pathogenic dataset (*k*=18, 22, 26 and 30), with and without filtering low complexity *k-*mers (entropy ≤2.5). The observation that some of the classification results did not agree with the RT-PCR findings is discussed in the main text. Notably, the classification was insensitive to the size of *k*, with only two samples (1B5 and 1F7) not yielding the same result at all four sizes. Moreover, entropy filtering only influenced one sample at *k*=18 (1F7), where the top hit with filtering matched the RT-PCR assignment (HRV-B) but another target (human betaherpesvirus 5) without filtering (Table S2). Taking into account the results of these experiments, a default *k*-mer size of 22 was built into DisCVR. At this value, the average execution time per sample was 1 m 47 s on a Windows OS machine with 32 GB of RAM.

| **Table S1**. Tri-nucleotide entropy values of repetitive and non-repetitive *k*-mers. | | |
| --- | --- | --- |
| *k* | *k*-mer sequence | Entropy |
| 18 | AAAAAAAAAAAAAAAAAA | 0.0 |
|  | TGTGTGTGTGTGTGTGTG | 1.0 |
|  | GGTCCAGTGAAAGATCCT | 2.6 |
| 22 | AAAAAAAAAAAAAAAAAAAAAA | 0.0 |
|  | TGTGTGTGTGTGTGTGTGTGTG | 1.0 |
|  | GGTCCAGTGAAAGATCCTGTCA | 2.8 |
| 26 | AAAAAAAAAAAAAAAAAAAAAAAAAA | 0.0 |
|  | TGTGTGTGTGTGTGTGTGTGTGTGTG | 1.0 |
|  | GGTCCAGTGAAAGATCCTGTCTATAG | 3.0 |
| 30 | AAAAAAAAAAAAAAAAAAAAAAAAAAAAAA | 0.0 |
|  | TGTGTGTGTGTGTGTGTGTGTGTGTGTGTG | 1.0 |
|  | GGTCCAGTGAAAGATCCTGTCTATAGCATA | 3.3 |

| 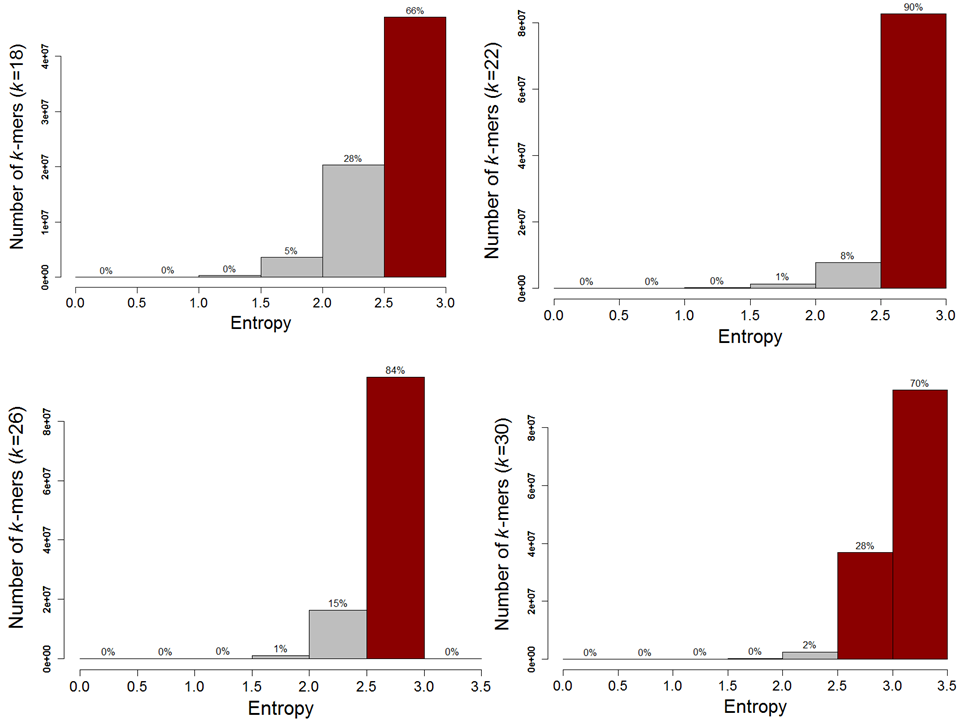 |
| --- |
| **Figure S1.** Entropy distribution for different values of k. k-mers above the threshold of 2.5 are indicated in red. |

| 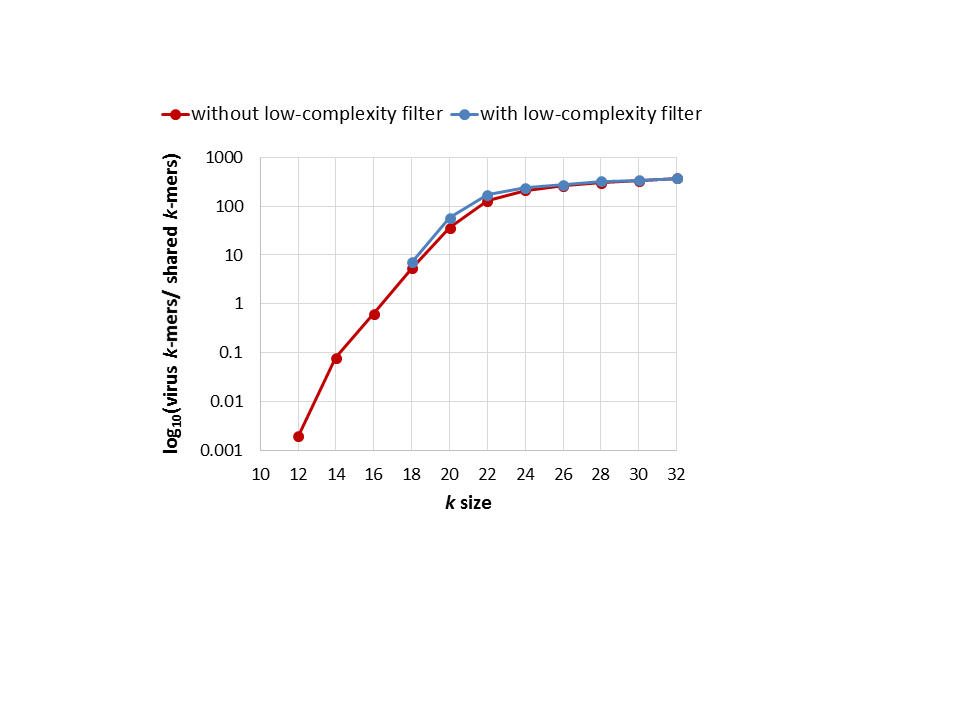 |
| --- |
| **Figure S2.** Relationship between the size of k and the proportion of virus k-mers in the pathogenic dataset. Low-complexity filter refers to the exclusion of low-complexity k-mers. All k-mers for k<18 were below the low-complexity threshold. |

| 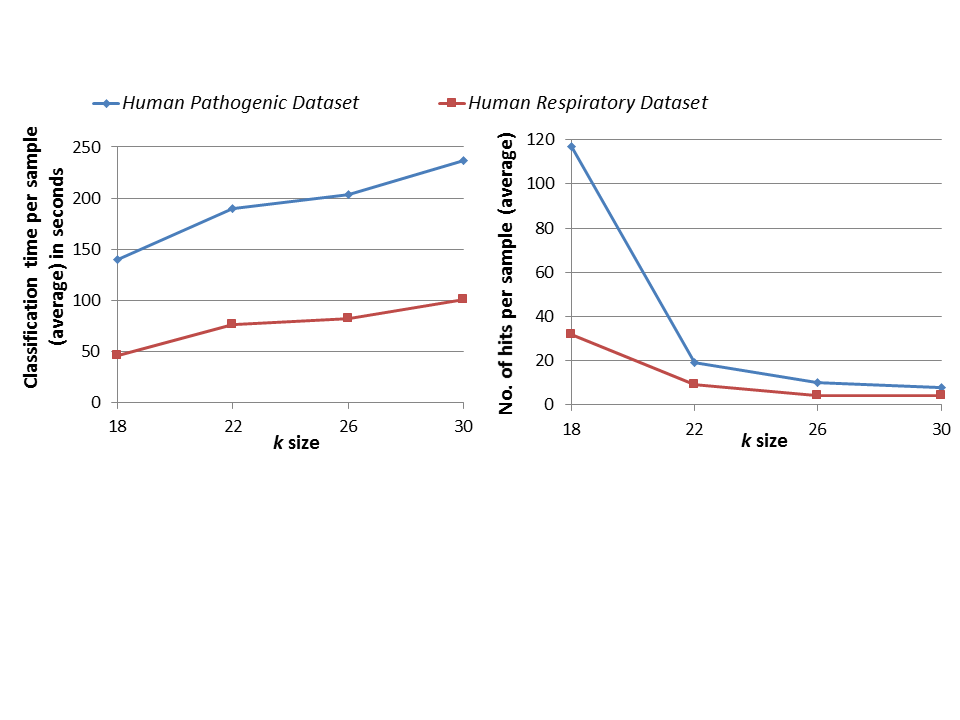 |
| --- |
| **Figure S3.** Relationship between k-mer size and classification results. |
